# Fast‐forwarding plant breeding with deep learning‐based genomic prediction

**DOI:** 10.1111/jipb.13914

**Published:** 2025-04-14

**Authors:** Shang Gao, Tingxi Yu, Awais Rasheed, Jiankang Wang, Jose Crossa, Sarah Hearne, Huihui Li

**Affiliations:** ^1^ State Key Laboratory of Crop Gene Resources and Breeding, Institute of Crop Sciences Chinese Academy of Agricultural Sciences, CIMMYT‐China office Beijing 100081 China; ^2^ Nanfan Research Institute Chinese Academy of Agricultural Sciences Sanya 572024 China; ^3^ Department of Plant Sciences Quaid‐i‐Azam University Islamabad 45320 Pakistan; ^4^ International Maize and Wheat Improvement Center (CIMMYT) Apdo. Postal 6‐641, Texcoco, D.F. 06600 Mexico

**Keywords:** artificial intelligence, deep learning, genomic prediction, plant breeding

## Abstract

Deep learning‐based genomic prediction (DL‐based GP) has shown promising performance compared to traditional GP methods in plant breeding, particularly in handling large, complex multi‐omics data sets. However, the effective development and widespread adoption of DL‐based GP still face substantial challenges, including the need for large, high‐quality data sets, inconsistencies in performance benchmarking, and the integration of environmental factors. Here, we summarize the key obstacles impeding the development of DL‐based GP models and propose future developing directions, such as modular approaches, data augmentation, and advanced attention mechanisms.

## Graphical abstract

Summary:

Deep learning‐based genomic prediction provides powerful tools for plant breeding through advanced architectures and large datasets. This review summarizes current progress and challenges in this rapidly evolving field, stressing the importance of modularized development, data augmentation, and standardized evaluation for developing future genomic prediction methods.

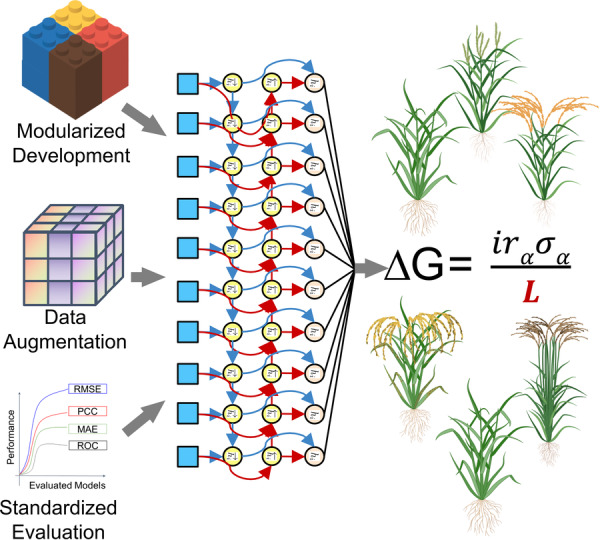

## BACKGROUND FOR GENOMIC PREDICTION TASKS IN PLANT BREEDING

Traditionally, plant breeding relies on phenotypic evaluation and marker‐assisted selection, which faces limitations in efficiently capturing relevant patterns from ever‐growing multi‐omics input data sets. Genomic prediction (GP) integrates molecular markers and aims to accurately predict phenotypic performance (Xu et al., 2022; Farooq et al., 2024). The efficiency of GP is known to be affected by factors such as the genetic architecture of target traits, population size, and the extent of linkage disequilibrium (among others). The selection of appropriate and informative models for GP analysis is recognized as an essential enabling step. In recent years, deep learning (DL)‐based models have shown competitive or even better performance than traditional linear regression models for big‐data‐driven GP, motivated by their capacity for automatic feature extraction and enhanced representation of high‐dimensional data sets.

In 2016, researchers applied a multi‐layer perceptron (MLP) model to plant predictive breeding, and such models have subsequently been used for GP in wheat and maize (González‐Camacho et al., 2016). However, owing to their relatively simple architecture, MLP models have consistently failed to achieve substantial improvements over traditional linear models. With the rapid development of DL and its applications in GP, various updated neural network architectures have emerged, achieving increasingly impressive performance.

## CURRENT DL‐BASED GP METHODS

Deep‐learning‐based GP methods in plant breeding have seen steady improvement driven by the rapid development of artificial intelligence (AI) techniques. The first DL models that brought substantial advancement to GP were convolutional neural networks (CNNs). Ma et al. (2018) developed a model for genomic selection, DeepGS, which employs a CNN architecture consisting of one convolutional layer, one sampling layer, three dropout layers, and two fully connected layers. The study reporting the model applied it to a 2,000‐individual wheat data set and employed a widely used linear method, ridge regression‐best linear unbiased prediction (RR‐BLUP), for performance benchmarking. DeepGS exhibited comparable accuracy to RR‐BLUP across eight tested traits, and showed distinct strengths in predicting high breeding value individuals compared to RR‐BLUP.

Deep‐learning models can handle diverse types of input data. Washburn et al. (2021) examined multiple CNN models developed for predicting maize yields from a combination of replicated trials and historical yield survey data. The model incorporating daily weather and historical survey data outperformed BLUP methods. Wang et al. (2023) developed DNNGP, a DL model integrating multi‐omics data, and it significantly outperformed traditional models, even when using single nucleotide polymorphism (SNP) data as the only input.

SoyDNGP introduced a coordinate attention module within a CNN architecture (Gao et al., 2023), which resulted in better performance than both traditional and DL‐based methods, with this improvement attributable the attention module's capacity to harness positional information. GPformer uses a transformer‐based structure for self‐attention, incorporating both one‐hot encoded base variation and physical position information for all variants (Wu et al., 2023) to identify feature representations from the genome‐wide level. Besides the improved prediction accuracy, these DL‐based GP methods overcome the drawbacks of extensive computational time required by the traditional methods, such as the Bayesian alphabet models, especially when dealing with populations over thousands of lines (Wang et al., 2023).

These recent advancements in DL‐based GP can be compared and categorized according to various development strategies and model architectures. Methods such as DeepGS (Ma et al., 2018), DNNGP (Wang et al., 2023), DeepCCR (Ma et al., 2024), and SoyDNGP (Gao et al., 2023), rely on CNN‐based architectures, which are particularly effective at extracting local patterns from structured genomic data such as SNP arrays. Meanwhile, transformer‐based models, such as GPformer (Wu et al., 2023) and Cropformer (Wang et al., 2025), enhance feature extraction by capturing long‐range dependencies among genetic variants, making them well‐suited for sequence data. Another category of models, including TrG2P (Li et al., 2024) and DEM (Ren et al., 2024), integrates multi‐trait and functional genomic information to refine predictions by leveraging biological priors or transfer learning strategies. These approaches improve model generalizability and facilitate gene discovery by extracting relevant genetic interactions. Furthermore, automated frameworks such as Auto‐GS (He et al., 2025) and AutoGP (Wu et al., 2025) have been developed to provide user‐friendly platforms for genomic selection, integrating multiple machine learning (ML) and DL models to enhance accessibility for breeders. Collectively, these innovations highlight the increasing adaptability of DL‐based GP in terms of different breeding programs, population structures, and data modalities.

## CHALLENGES FOR DEVELOPING DL‐BASED GP MODELS

Despite their preliminary success, DL‐based GP methods still face challenges. One notable limitation is the dependency on large data sets. Deep‐learning methods in plant breeding have often been limited to small data sets with sizes typically ranging from several hundred to no more than 1,000 lines. Wang et al. (2023) examined the impact of population size gradients on prediction accuracy of DNNGP in a 2,000‐individual wheat population, and found that larger populations correlated with enhanced prediction accuracy across all traits. Montesinos‐López et al. (2024) examined the performance of a multimodal DL model with a moderately large data set comprising 4,464 wheat lines, and achieved higher accuracy than GBLUP for multiple traits. Another sample size‐gradient test on SoyDNGP—conducted on more than 10,000 soybean lines—found that the prediction accuracies for both regressors and classifiers were gradually improved as the training sample size increased. It seems likely that DL‐based GP stands to benefit from gains in performance as data set size increases, similar to reports from fields including image processing, semantic information mining, and protein structure prediction.

From another perspective, the increase of data sets size holds the promise to train models with millions to billions of parameters. Foundation models, featured by ChatGPT, are those trained on massive data sets encompassing diverse scenarios and then fine‐tuned on smaller, specific data sets. This approach could be adapted for plant breeding, where a large‐scale, unsupervised learning phase on extensive genomic and phenotypic data is followed by targeted fine‐tuning using breeder‐specific experiments. Such a strategy would enable the development of robust, generalized models that can then be customized for particular breeding programs.

Multiple studies have reported that the inclusion of environmental factors (E) and genotype–environment interaction (*G* × *E*) information can substantially improve GP predictive ability (Xu et al., 2022; He et al., 2025; Wang et al., 2025). Deep‐learning models present a promising avenue to harness environmental data in diverse ways. It seems plausible that multiple strategies for integrating environmental factors could boost the performance of GP models, including feature engineering to extract relevant environmental features, data augmentation techniques to enrich the diversity of environmental data (e.g., generative adversarial networks, gaussian noise adding), and design of model architectures optimized for handling multimodal inputs (e.g., graph neural networks, transformers with cross‐attention). We take the view that integration of environmental data into GP will be a particularly profitable direction for the future development of DL‐based GP tools.

A common challenge in DL (and indeed many areas of computer science research) is inconsistencies in the performance benchmarking approaches used for the same model in different studies. For instance, despite examining the same wheat data set, there was an obvious discrepancy in the reported performance of DNNGP in two studies (Gao et al., 2023; Wang et al., 2023). We advocate that authors reporting on DL‐based GP methods should provide details such as the relevant hyperparameter settings, model configuration environment, training framework, and source code to enhance model reproducibility and transparency. The discrepancy reported in the two studies examining DNNGP reflect different metrics for measuring prediction accuracy and parameter tuning (with highly divergent computational resource investment). As the application of DL for GP continues to expand, inconsistencies in performance benchmarking methods will likely confuse users and impede research and application progress. This situation could be ameliorated through the development and promulgation of a standardized DL model evaluation framework, including (but not limited to) a hyperparameter optimization protocol and use of agreed‐upon criteria and metrics for model assessment.

## AN APPROACH FOR THE RATIONAL DEVELOPMENT OF DL‐BASED GP MODELS

**Figure 1:**
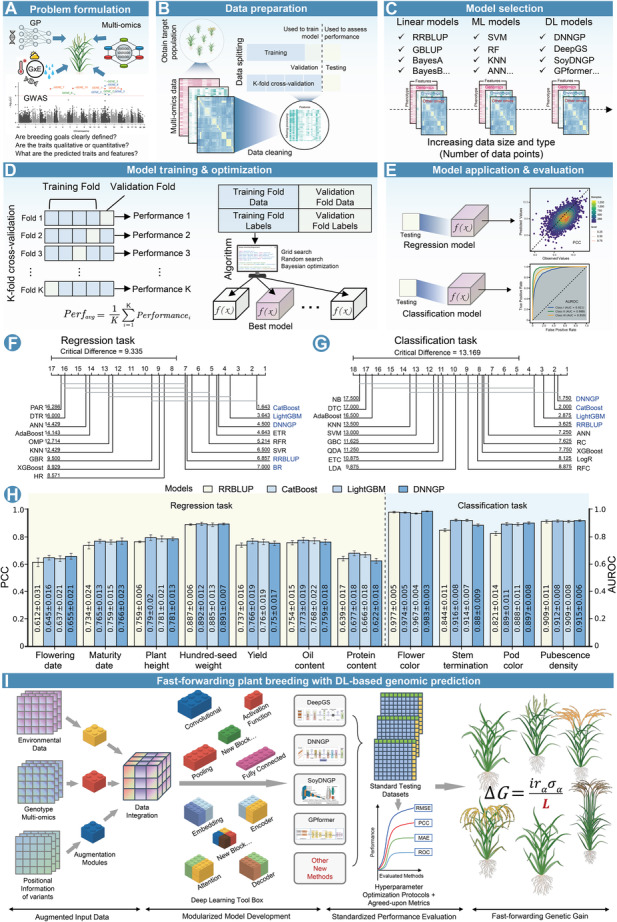
Workflow of developing deep learning‐based genomic prediction (DL‐based GP) models and fast‐forwarding plant breeding driven by these models **(A)** Define the research question and prediction task. **(B)** Collect, preprocess, and split data. **(C)** Select the appropriate model based on the prediction task and data set. **(D)** Train and optimize the model using *K*‐fold cross‐validation. **(E)** Test and evaluate the model. **(F)** and **(G)** are comparisons of regression and classification models’ performances by critical difference diagram for multiple regression and classification tasks. The horizontal axis represents the average rank of the models, where a lower rank indicates better performance. Groups of models with rank differences smaller than the critical difference score are connected by gray lines, indicating no significant difference on performance (*P* = 0.05). **(H)** Classification and regression performance comparison for DNNGP, RR‐BLUP, CatBoost, and LightGBM on 11 traits. **(I)** Fast‐forwarding plant breeding with DL‐based genomic prediction. Regarding DL‐based genomic prediction, we envision a future where three trends can converge to expedite crop improvement. First, by amplifying our data sets through data augmentation, users can enrich training samples with diverse genotypic, phenotypic, and environmental data. Second, adopting a modular approach to model development—akin to assembling LEGO® blocks—allows developers to iteratively enhance individual model components and reintegrate them into mature architectures. Third, standardizing model components across different data types should streamline development, fostering collaboration, and reducing barriers within the plant‐breeding community. As artificial intelligence (AI) research in plant biology accelerates, active collaboration between breeders and AI experts should help to unlock the full potential of DL‐driven crop breeding. The breeder's equation in the rightmost panel illustrates how genetic gain is determined by *i*: selection intensity, *r*_*α*_: genetic correlation/selection accuracy, *σ*_*α*_: standard deviation of breeding values, and L: generation interval. Applying a DL‐based GP method would shorten the generation interval to fast‐forward genetic gain in plant breeding.

When developing DL methods for GP, it is advisable to initially fully understand the input data and the prediction task, and focus on traditional ML and statistical models as the primary exploratory tools, and use them as benchmark models for comparison with DL models, to identify the most suitable solutions for a given task (Smith et al., 2020; Greener et al., 2022; Yang et al., 2023; Chen et al., 2024; Huang et al., 2024). Given the plethora of models available for classification and regression tasks, adopting an empirical and trial‐and‐error approach to identify the best model during the initial phase is often the most cautious strategy (Smith et al., 2020). Therefore, a sound strategy for developing and selecting the optimal model involves training and optimizing the various methods mentioned, choosing the one that performs best on the validation set, and ultimately evaluating their performance using an independent test set (Figure 1).

In the process of model training, the data is divided into training, validation, and test sets. The training set is used to update model parameters, while the validation set monitors the training process, selects hyperparameters, and prevents overfitting. It is crucial to ensure that there is no data leakage between the training and test sets, and the test set should be sufficiently large to provide reliable results while reflecting real‐world scenarios in future applications (Figure 1B, C). Moreover, the model training process needs to be tuned through methods such as random search, grid search, or Bayesian optimization combined with *K*‐fold cross‐validation, using a validation set to monitor for potential overfitting without interfering with training (Bzdok et al., 2018) (Figure 1D). Finally, the test set is used to evaluate the model's performance on data that were not involved in training or validation, providing an objective measure of the model's generalization ability (Figure 1E).

Model selection depends on the prediction task, technological trends, and the suitability of the data. The use of a DL model should stem from the specific needs of the data and the problem at hand, rather than simply imitating other studies. At this stage, the best practice is to compare the new model with existing models that have demonstrated good performance, such as the statistical model RR‐BLUP, ML‐based methods like CatBoost and LightGBM, and DL‐based methods like DNNGP and SoyDNGP. Comparing various model types can provide a more comprehensive interpretation of the results. Additionally, during the model evaluation process, it is advisable to select appropriate evaluation metrics based on the nature and significance of the tasks or to combine multiple metrics to assess the model's overall performance.

We provide a case study using a soybean data set containing 20,087 soybean accessions and 42,509 high‐confidence SNPs (Gao et al., 2023) to illustrate the selection and training process of comparing models. In the initial model selection stage, we selected 31 classification and regression models from the ML library to serve as benchmark test models for classification and regression tasks in genome selection (Table S1). Then, we used the critical difference diagram (Demšar, 2006) to evaluate the performance of these models on the classification and regression tasks on multiple traits, prioritized the models based on the evaluation results, and finally selected the better‐performing models as benchmarks (Figure 1F, G; Tables S2, S3). For further optimization training, we selected the top two ranked ML models CatBoost and LightGBM as well as a statistical model RR‐BLUP, and compared their performance with that of a DNNGP model (Figure 1H). The results show that the DNNGP model outperforms the optimal ML models CatBoost and LightGBM as well as the statistical model RR‐BLUP in terms of prediction performance in the classification task. In the regression task, DNNGP performs comparably with the combined performance of these three models.

## EXPANDING GP: INTEGRATING DL‐BASED GENE DISCOVERY

Gene discovery can enhance our understanding of the biological basis of complex traits, providing an essential foundation for guiding rational molecular design and intelligent breeding strategies. Deep‐learning‐based gene discovery leverages DL algorithms to identify functional genes and genetic interactions from large‐scale genomic and multi‐omics data, enabling a more precise understanding of complex traits. Innovative DL‐based gene discovery methods offer a promising avenue for enhancing GP in plant breeding. By harnessing genes and genetic interaction networks associated with target traits, the predictive capabilities of GP models can be improved (Xu et al., 2022; Wu et al., 2023). Recent studies have used DL methods to explore salt stress‐related genes and genetic networks in plants (Yang et al., 2023; Chen et al., 2024; Huang et al., 2024). These studies identified relevant genes that can potentially be integrated into GP models as weight layers or alternative input data to tune model performance for GP of related traits. Looking ahead, collaborative efforts between researchers from ML and plant genomics and breeding can advance the integration of gene discovery into GP. As with the aforementioned opportunity with hyperparameter optimization, it seems virtually certain that development of standardized frameworks and protocols for incorporating discovered genes and genetic networks will facilitate the development of effective GP models for specific traits across diverse species and breeding programs.

## CONCLUSIONS AND OUTLOOK

Deep‐learning models are modular and have been conceptualized as being “LEGO®‐like”; that is, enabling the addition of new functional modules into mature architectures to improve model performance (Alom et al., 2019). And we also know that the performance of individual modules can be upgraded individually and then reintroduced into model architectures. To enhance DL models for GP, we propose two urgently needed “LEGO® blocks”: data augmentation and advanced attention mechanisms (Figure 1I). Data augmentation enriches training data sets with diverse genotypic, phenotypic, and environmental data. Integrating advanced attention mechanisms, such as self‐attention and/or cross‐attention, can be expected to enhance a model's ability to focus on relevant features across different data types. Standardizing DL modules for processing particular data types should streamline model development and thereby reduce barriers to their development and practical use by the plant breeding community.

As the application of AI tools continues to evolve in plant breeding, we anticipate a growing convergence between DL‐based GP and other emerging fields, such as synthetic biology, gene editing, and high‐throughput phenotyping. It seems quite obvious that the integration of AI tools into plant breeding practices holds promise for shortening the generation interval, and therefore accelerating trait improvement. However, to fully realize the potential of DL‐based GP in plant breeding, it is essential to foster broader industry engagement and enhance cross‐disciplinary collaborations. The development and application of DL models across various stages of plant breeding require contributions from experts in fields such as agriculture, computational biology and ML. A more extensive, integrated effort across sectors will help DL‐based GP methods thrive, ensuring they can be applied quickly and effectively in diverse breeding programs.

International collaboration is also important for the continued success of DL‐based GP development, particularly given the essential need for large‐scale, high‐quality data. Strengthening global partnerships can facilitate the integration of large‐scale field trials to generate more comprehensive breeding data sets, which is the foundation for developing better DL‐based GP models. Such collaborations would also enable worldwide experts to work together on algorithm innovations, ultimately advancing the pace of DL adoption in plant breeding. By sharing data and expertise across borders, we would be able to develop more robust, generalized GP methods capable of tackling a wider array of challenges faced by breeders globally.

Looking ahead, the future of AI‐driven plant breeding will rely on these collaborative efforts, not just within individual countries, but globally, to unlock the true potential of GP models. As we continue to push the boundaries of technology, the role of interdisciplinary cooperation and international partnerships will be central to accelerating the application of DL algorithms in plant breeding and advancing crop improvement worldwide.

## CONFLICTS OF INTEREST

The authors declare no competing interests.

## AUTHOR CONTRIBUTIONS

H.L. conceived the original idea, and designed the work. S.G. and H.L. drafted the manuscript. T.Y. conducted data analyses. A.R., J.W., J.C., and S.H. provide constructive advice on experimental design and results interpretation. All authors read and approved the final manuscript.

## Supporting information

Additional Supporting Information may be found online in the supporting information tab for this article: http://onlinelibrary.wiley.com/doi/10.1111/jipb.13914/suppinfo

**Table S1.** A total of 31 regression and classification models were trained as benchmark models for the development of deep learning‐based genomic prediction (DL‐based GP)
**Table S2.** The comparison of prediction performance among ANN, LogR, SVR, RC, LDA, SVM, CatBoost, XGBoost, LightGBM, QDA, GBC, RFC, KNN, ETC, AdaBoost, NB, and DTC for four qualitative traits in soybean was conducted
**Table S3.** The comparison of prediction performance among CatBoost, ETR, LightGBM, RFR, BR, XGBoost, HR, GBR, KNN, ANN, OMP, SVR, AdaBoost, DTR, and PAR for seven quantitative traits in soybean was conducted
