# Supplementary material for: Fast‐forwarding plant breeding with deep learning‐based genomic prediction
Source: J Integr Plant Biol. 2025 Apr 14;67(7):1700–5. doi: 10.1111/jipb.13914 (PMC12225013; doi:10.1111/jipb.13914)
Supplement: Supplementary file 1 — Table S1. A total of 31 regression and classification models were trained as benchmark models for the development of deep learning‐based genomic prediction (DL‐based GP) Table S2. The comparison of prediction performance among ANN, LogR, SVR, RC, LDA, SVM, CatBoost, XGBoost, LightGBM, QDA, GBC, RFC, KNN, ETC, AdaBoost, NB, and DTC for four qualitative traits in soybean was conducted Table S3. The comparison of prediction performance among CatBoost, ETR, LightGBM, RFR, BR, XGBoost, HR, GBR, KNN, ANN, OMP, SVR, AdaBoost, DTR, and PAR for seven quantitative traits in soybean was conducted [file JIPB-67-1700-s001.docx]

**Supplementary information**

**Fast-forwarding Plant Breeding with Deep Learning-based Genomic Prediction**

Shang Gao, Tingxi Yu, Awais Rasheed, Jiankang Wang, Jose Crossa, Sarah Hearne, Huihui Li^*^

**Supplementary Tables S1-S3**

**Table S1** A total of 31 regression and classification models were trained as benchmark models for the development of DL-based GP.

| **Model** | **Model description** |
| --- | --- |
| **Regression prediction** |  |
| CatBoost | Categorical Boosting |
| LightGBM | Light Gradient Boosting Machine |
| XGBoost | Extreme Gradient Boosting |
| AdaBoost | Adaptive Boosting |
| ETR | Extra Trees Regressor |
| RFR | Random Forest Regressor |
| BR | Bayesian Ridge |
| SVR | Support Vector Regression |
| HR | Huber Regressor |
| GBR | Gradient Boosting Regressor |
| KNN | K-Nearest Neighbor |
| OMP | Orthogonal Matching Pursuit |
| ANN | Artificial Neural Networks |
| DTR | Decision Tree Regressor |
| PAR | Passive Aggressive Regressor |
| **Classification prediction** |  |
| XGBoost | Extreme Gradient Boosting |
| CatBoost | Categorical Boosting |
| LightGBM | Light Gradient Boosting Machine |
| AdaBoost | Adaptive Boosting |
| ANN | Artificial Neural Networks |
| RC | Ridge Classifier |
| LogR | Logistic Regression |
| RFC | Random Forest Classifier |
| LDA | Linear Discriminant Analysis |
| ETC | Extra Trees Classifier |
| QDA | Quadratic Discriminant Analysis |
| GBC | Gradient Boosting Classifier |
| SVM | Support Vector Machine |
| KNN | K-Nearest Neighbor |
| DTC | Decision Tree Classifier |
| NB | Naive Bayes |

| **Table S2 (ST)** The comparison of prediction performance among ANN, LogR, SVR, RC, LDA, SVM, CatBoost, XGBoost, LightGBM, QDA, GBC, RFC, KNN, ETC, AdaBoost, NB, and DTC for four qualitative traits in soybean was conducted. The prediction performance of each method was evaluated using the Accuracy, AUROC, Recall, Precision, F1, Kappa, and MCC metrics. All models were trained using 10-fold cross-validation. | | | | | | | | |
| --- | --- | --- | --- | --- | --- | --- | --- | --- |
| **Model** | **Model description** | **Accuracy** | **AUROC** | **Recall** | **Precision** | **F1** | **Kappa** | **MCC** |
| XGBoost | Extreme Gradient Boosting | 0.817 | 0.912 | 0.817 | 0.801 | 0.804 | 0.662 | 0.665 |
| CatBoost | CatBoost Classifier | 0.816 | 0.916 | 0.816 | 0.800 | 0.800 | 0.659 | 0.662 |
| LightGBM | Light Gradient Boosting Machine | 0.815 | 0.914 | 0.815 | 0.798 | 0.800 | 0.657 | 0.661 |
| RC | Ridge Classifier | 0.811 | 0.901 | 0.811 | 0.791 | 0.789 | 0.645 | 0.651 |
| RF | Random Forest Classifier | 0.811 | 0.902 | 0.811 | 0.794 | 0.794 | 0.649 | 0.652 |
| LogR | Logistic Regression | 0.807 | 0.906 | 0.807 | 0.790 | 0.794 | 0.642 | 0.646 |
| ETC | Extra Trees Classifier | 0.807 | 0.891 | 0.807 | 0.788 | 0.792 | 0.642 | 0.645 |
| GBC | Gradient Boosting Classifier | 0.805 | 0.905 | 0.805 | 0.789 | 0.784 | 0.635 | 0.641 |
| LDA | Linear Discriminant Analysis | 0.804 | 0.905 | 0.804 | 0.797 | 0.800 | 0.645 | 0.646 |
| ANN | Artificial Neural Networks | 0.802 | 0.900 | 0.802 | 0.791 | 0.795 | 0.640 | 0.641 |
| QDA | Quadratic Discriminant Analysis | 0.785 | 0.887 | 0.785 | 0.792 | 0.787 | 0.622 | 0.623 |
| SVM | SVM - Linear Kernel | 0.784 | 0.885 | 0.784 | 0.767 | 0.773 | 0.603 | 0.605 |
| KNN | K Neighbors Classifier | 0.776 | 0.872 | 0.776 | 0.764 | 0.768 | 0.590 | 0.592 |
| AdaBoost | AdaBoost Classifier | 0.763 | 0.853 | 0.763 | 0.739 | 0.746 | 0.560 | 0.563 |
| DTC | Decision Tree Classifier | 0.729 | 0.769 | 0.729 | 0.731 | 0.730 | 0.518 | 0.518 |
| NB | Naive Bayes | 0.697 | 0.827 | 0.697 | 0.717 | 0.702 | 0.478 | 0.483 |
| **ST:** stem termination | |  |  |  |  |  |  |  |
| **Accuracy**: Accuracy | |  |  |  |  |  |  |  |
| **AUROC**: Area Under the Receiver Operating Characteristic Curve | | | | |  |  |  |  |
| **Recall**: Recall | |  |  |  |  |  |  |  |
| **Precision**: Precision | |  |  |  |  |  |  |  |
| **F1**: F1 Score | |  |  |  |  |  |  |  |
| **Kappa**: Cohen’s Kappa | |  |  |  |  |  |  |  |
| **MCC**: Matthews Correlation Coefficient | |  |  |  |  |  |  |  |

| **Table S2 (FC)** The comparison of prediction performance among ANN, LogR, SVR, RC, LDA, SVM, CatBoost, XGBoost, LightGBM, QDA, GBC, RFC, KNN, ETC, AdaBoost, NB, and DTC for four qualitative traits in soybean was conducted. The prediction performance of each method was evaluated using the Accuracy, AUROC, Recall, Precision, F1, Kappa, and MCC metrics. All models were trained using 10-fold cross-validation. | | | | | | | | |
| --- | --- | --- | --- | --- | --- | --- | --- | --- |
| **Model** | **Model description** | **Accuracy** | **AUROC** | **Recall** | **Precision** | **F1** | **Kappa** | **MCC** |
| ANN | Artificial Neural Networks | 0.953 | 0.984 | 0.929 | 0.931 | 0.930 | 0.895 | 0.895 |
| LogR | Logistic Regression | 0.947 | 0.980 | 0.919 | 0.920 | 0.919 | 0.879 | 0.879 |
| RC | Ridge Classifier | 0.943 | 0.905 | 0.915 | 0.915 | 0.915 | 0.872 | 0.872 |
| LDA | Linear Discriminant Analysis | 0.943 | 0.979 | 0.917 | 0.911 | 0.914 | 0.871 | 0.871 |
| SVM | SVM - Linear Kernel | 0.936 | 0.967 | 0.903 | 0.903 | 0.903 | 0.855 | 0.855 |
| CatBoost | CatBoost Classifier | 0.930 | 0.974 | 0.849 | 0.934 | 0.889 | 0.839 | 0.841 |
| XGBoost | Extreme Gradient Boosting | 0.921 | 0.971 | 0.839 | 0.917 | 0.876 | 0.818 | 0.820 |
| LightGBM | Light Gradient Boosting Machine | 0.916 | 0.967 | 0.826 | 0.913 | 0.867 | 0.806 | 0.809 |
| QDA | Quadratic Discriminant Analysis | 0.890 | 0.937 | 0.837 | 0.833 | 0.835 | 0.752 | 0.752 |
| GBC | Gradient Boosting Classifier | 0.884 | 0.949 | 0.737 | 0.895 | 0.808 | 0.726 | 0.733 |
| RF | Random Forest Classifier | 0.874 | 0.951 | 0.657 | 0.946 | 0.775 | 0.692 | 0.714 |
| KNN | K Neighbors Classifier | 0.857 | 0.902 | 0.756 | 0.800 | 0.777 | 0.672 | 0.672 |
| ETC | Extra Trees Classifier | 0.856 | 0.951 | 0.599 | 0.949 | 0.734 | 0.643 | 0.675 |
| AdaBoost | AdaBoost Classifier | 0.848 | 0.912 | 0.726 | 0.799 | 0.760 | 0.650 | 0.652 |
| NB | Naive Bayes | 0.819 | 0.895 | 0.675 | 0.753 | 0.712 | 0.580 | 0.582 |
| DTC | Decision Tree Classifier | 0.802 | 0.776 | 0.698 | 0.703 | 0.700 | 0.552 | 0.553 |
| **FC:** flower color | |  |  |  |  |  |  |  |
| **Accuracy**: Accuracy | |  |  |  |  |  |  |  |
| **AUROC:** Area Under the Receiver Operating Characteristic Curve | | | | |  |  |  |  |
| **Recall**: Recall | |  |  |  |  |  |  |  |
| **Precision**: Precision | |  |  |  |  |  |  |  |
| **F1**: F1 Score | |  |  |  |  |  |  |  |
| **Kappa**: Cohen’s Kappa | |  |  |  |  |  |  |  |
| **MCC**: Matthews Correlation Coefficient | |  |  |  |  |  |  |  |

| **Table S2 (POD)** The comparison of prediction performance among ANN, LogR, SVR, RC, LDA, SVM, CatBoost, XGBoost, LightGBM, QDA, GBC, RFC, KNN, ETC, AdaBoost, NB, and DTC for four qualitative traits in soybean was conducted. The prediction performance of each method was evaluated using the Accuracy, AUROC, Recall, Precision, F1, Kappa, and MCC metrics. All models were trained using 10-fold cross-validation. | | | | | | | | |
| --- | --- | --- | --- | --- | --- | --- | --- | --- |
| **Model** | **Model description** | **Accuracy** | **AUROC** | **Recall** | **Precision** | **F1** | **Kappa** | **MCC** |
| CatBoost | CatBoost Classifier | 0.825 | 0.890 | 0.825 | 0.826 | 0.814 | 0.580 | 0.596 |
| LightGBM | Light Gradient Boosting Machine | 0.822 | 0.888 | 0.822 | 0.823 | 0.810 | 0.570 | 0.587 |
| ANN | Artificial Neural Networks | 0.822 | 0.878 | 0.822 | 0.818 | 0.818 | 0.600 | 0.602 |
| XGBoost | Extreme Gradient Boosting | 0.820 | 0.888 | 0.820 | 0.818 | 0.808 | 0.568 | 0.582 |
| RF | Random Forest Classifier | 0.814 | 0.879 | 0.814 | 0.824 | 0.795 | 0.530 | 0.566 |
| QDA | Quadratic Discriminant Analysis | 0.808 | 0.838 | 0.808 | 0.811 | 0.802 | 0.569 | 0.570 |
| ETC | Extra Trees Classifier | 0.806 | 0.878 | 0.806 | 0.818 | 0.784 | 0.505 | 0.547 |
| KNN | K Neighbors Classifier | 0.804 | 0.851 | 0.804 | 0.799 | 0.799 | 0.555 | 0.558 |
| RC | Ridge Classifier | 0.802 | 0.726 | 0.802 | 0.797 | 0.789 | 0.526 | 0.539 |
| LogR | Logistic Regression | 0.801 | 0.861 | 0.801 | 0.795 | 0.794 | 0.542 | 0.548 |
| GBC | Gradient Boosting Classifier | 0.801 | 0.860 | 0.801 | 0.803 | 0.782 | 0.504 | 0.531 |
| LDA | Linear Discriminant Analysis | 0.800 | 0.860 | 0.800 | 0.795 | 0.794 | 0.543 | 0.548 |
| SVM | SVM - Linear Kernel | 0.778 | 0.907 | 0.778 | 0.772 | 0.773 | 0.500 | 0.503 |
| DTC | Decision Tree Classifier | 0.734 | 0.714 | 0.734 | 0.735 | 0.734 | 0.425 | 0.426 |
| NB | Naive Bayes | 0.722 | 0.781 | 0.722 | 0.728 | 0.720 | 0.392 | 0.395 |
| AdaBoost | AdaBoost Classifier | 0.721 | 0.666 | 0.721 | 0.697 | 0.694 | 0.304 | 0.322 |
| **POD:** pod color | |  |  |  |  |  |  |  |
| **Accuracy**: Accuracy | |  |  |  |  |  |  |  |
| **AUROC:** Area Under the Receiver Operating Characteristic Curve | | | | |  |  |  |  |
| **Recall**: Recall | |  |  |  |  |  |  |  |
| **Precision**: Precision | |  |  |  |  |  |  |  |
| **F1**: F1 Score | |  |  |  |  |  |  |  |
| **Kappa**: Cohen’s Kappa | |  |  |  |  |  |  |  |
| **MCC**: Matthews Correlation Coefficient | |  |  |  |  |  |  |  |

| **Table S2 (P_DENS)** The comparison of prediction performance among ANN, LogR, SVR, RC, LDA, SVM, CatBoost, XGBoost, LightGBM, QDA, GBC, RFC, KNN, ETC, AdaBoost, NB, and DTC for four qualitative traits in soybean was conducted. The prediction performance of each method was evaluated using the Accuracy, AUROC, Recall, Precision, F1, Kappa, and MCC metrics. All models were trained using 10-fold cross-validation. | | | | | | | | |
| --- | --- | --- | --- | --- | --- | --- | --- | --- |
| **Model** | **Model description** | **Accuracy** | **AUROC** | **Recall** | **Precision** | **F1** | **Kappa** | **MCC** |
| LightGBM | Light Gradient Boosting Machine | 0.840 | 0.909 | 0.751 | 0.831 | 0.789 | 0.661 | 0.663 |
| CatBoost | CatBoost Classifier | 0.839 | 0.912 | 0.746 | 0.833 | 0.787 | 0.658 | 0.661 |
| LogR | Logistic Regression | 0.838 | 0.906 | 0.775 | 0.809 | 0.792 | 0.659 | 0.659 |
| RC | Ridge Classifier | 0.838 | 0.903 | 0.769 | 0.814 | 0.790 | 0.658 | 0.659 |
| LDA | Linear Discriminant Analysis | 0.837 | 0.906 | 0.770 | 0.812 | 0.791 | 0.658 | 0.659 |
| ANN | Artificial Neural Networks | 0.836 | 0.907 | 0.780 | 0.803 | 0.791 | 0.656 | 0.656 |
| RF | Random Forest Classifier | 0.831 | 0.898 | 0.705 | 0.844 | 0.768 | 0.636 | 0.643 |
| XGBoost | Extreme Gradient Boosting | 0.830 | 0.902 | 0.748 | 0.811 | 0.778 | 0.641 | 0.643 |
| ETC | Extra Trees Classifier | 0.828 | 0.888 | 0.699 | 0.843 | 0.764 | 0.631 | 0.638 |
| GBC | Gradient Boosting Classifier | 0.819 | 0.894 | 0.706 | 0.814 | 0.756 | 0.613 | 0.617 |
| QDA | Quadratic Discriminant Analysis | 0.818 | 0.880 | 0.789 | 0.763 | 0.776 | 0.623 | 0.623 |
| SVM | SVM - Linear Kernel | 0.807 | 0.925 | 0.754 | 0.760 | 0.757 | 0.597 | 0.597 |
| KNN | K Neighbors Classifier | 0.800 | 0.867 | 0.772 | 0.739 | 0.755 | 0.587 | 0.587 |
| AdaBoost | AdaBoost Classifier | 0.792 | 0.859 | 0.690 | 0.765 | 0.725 | 0.558 | 0.560 |
| DTC | Decision Tree Classifier | 0.754 | 0.743 | 0.689 | 0.691 | 0.690 | 0.486 | 0.486 |
| NB | Naive Bayes | 0.726 | 0.775 | 0.660 | 0.655 | 0.657 | 0.429 | 0.429 |
| **P_DENS:** pubescence density | |  |  |  |  |  |  |  |
| **Accuracy**: Accuracy | |  |  |  |  |  |  |  |
| **AUROC:** Area Under the Receiver Operating Characteristic Curve | | | | |  |  |  |  |
| **Recall**: Recall | |  |  |  |  |  |  |  |
| **Precision**: Precision | |  |  |  |  |  |  |  |
| **F1**: F1 Score | |  |  |  |  |  |  |  |
| **Kappa**: Cohen’s Kappa | |  |  |  |  |  |  |  |
| **MCC**: Matthews Correlation Coefficient | |  |  |  |  |  |  |  |

| **Table S3 (Yield)** The comparison of prediction performance among ANN, LogR, SVR, RC, LDA, SVM, CatBoost, XGBoost, LightGBM, QDA, GBC, RFC, KNN, ETC, AdaBoost, NB, and DTC for four qualitative traits in soybean was conducted. The prediction performance of each method was evaluated using the Accuracy, AUROC, Recall, Precision, F1, Kappa, and MCC metrics. All models were trained using 10-fold cross-validation. | | | | | | | | |
| --- | --- | --- | --- | --- | --- | --- | --- | --- |
| **Model** | **Model description** | **MAE** | **MSE** | **RMSE** | **R2** | **RMSLE** | **MAPE** | **PCC** |
| SVR | Support Vector Regression | 0.358 | 0.213 | 0.461 | 0.603 | 0.168 | 0.254 | 0.777 |
| CatBoost | CatBoost Regressor | 0.369 | 0.222 | 0.471 | 0.585 | 0.173 | 0.265 | 0.766 |
| LightGBM | Light Gradient Boosting Machine | 0.374 | 0.227 | 0.476 | 0.576 | 0.175 | 0.271 | 0.760 |
| ETR | Extra Trees Regressor | 0.373 | 0.229 | 0.479 | 0.571 | 0.176 | 0.268 | 0.756 |
| RFR | Random Forest Regressor | 0.378 | 0.232 | 0.482 | 0.566 | 0.177 | 0.275 | 0.753 |
| BR | Bayesian Ridge | 0.390 | 0.245 | 0.495 | 0.543 | 0.182 | 0.284 | 0.737 |
| HR | Huber Regressor | 0.390 | 0.247 | 0.497 | 0.538 | 0.182 | 0.283 | 0.735 |
| XGBoost | Extreme Gradient Boosting | 0.387 | 0.248 | 0.498 | 0.536 | 0.181 | 0.272 | 0.736 |
| GBR | Gradient Boosting Regressor | 0.403 | 0.256 | 0.506 | 0.523 | 0.187 | 0.304 | 0.727 |
| KNN | K Neighbors Regressor | 0.400 | 0.269 | 0.519 | 0.497 | 0.189 | 0.288 | 0.708 |
| OMP | Orthogonal Matching Pursuit | 0.424 | 0.280 | 0.529 | 0.477 | 0.195 | 0.315 | 0.691 |
| ANN | Artificial Neural Networks | 0.453 | 0.347 | 0.589 | 0.350 | 0.213 | 0.302 | 0.668 |
| AdaBoost | AdaBoost Regressor | 0.461 | 0.319 | 0.564 | 0.406 | 0.209 | 0.355 | 0.645 |
| PAR | Passive Aggressive Regressor | 0.515 | 0.430 | 0.655 | 0.198 | 0.238 | 0.351 | 0.589 |
| DTR | Decision Tree Regressor | 0.515 | 0.457 | 0.676 | 0.145 | 0.247 | 0.351 | 0.579 |
| **Yield:**Yield |  |  |  |  |  |  |  |  |
| **MAE**: Mean Absolute Error | |  |  |  |  |  |  |  |
| **MSE**: Mean Squared Error | |  |  |  |  |  |  |  |
| **RMSE**: Root Mean Squared Error | |  |  |  |  |  |  |  |
| **R²**: R-squared (Coefficient of Determination) | |  |  |  |  |  |  |  |
| **RMSLE**: Root Mean Squared Logarithmic Error | | |  |  |  |  |  |  |
| **MAPE**: Mean Absolute Percentage Error | |  |  |  |  |  |  |  |
| **PCC**: Pearson Correlation Coefficient | |  |  |  |  |  |  |  |

| **Table S3 (SdWgt)** The comparison of prediction performance among CatBoost, ETR, LightGBM, RFR, BR, XGBoost, HR, GBR, KNN, ANN, OMP, SVR, AdaBoost, DTR, and PAR for seven quantitative traits in soybean was conducted. The prediction performance of each method was evaluated using the MAE, MSE, RMSE, R2, RMSLE, MAPE, and PCC metrics. All models were trained using 10-fold cross-validation. | | | | | | | | |
| --- | --- | --- | --- | --- | --- | --- | --- | --- |
| **Model** | **Model description** | **MAE** | **MSE** | **RMSE** | **R2** | **RMSLE** | **MAPE** | **PCC** |
| CatBoost | CatBoost Regressor | 1.724 | 5.871 | 2.422 | 0.795 | 0.148 | 0.120 | 0.892 |
| SVR | Support Vector Regression | 1.828 | 6.511 | 2.550 | 0.773 | 0.157 | 0.129 | 0.892 |
| BR | Bayesian Ridge | 1.835 | 6.130 | 2.475 | 0.786 | 0.155 | 0.128 | 0.887 |
| HR | Huber Regressor | 1.819 | 6.152 | 2.479 | 0.785 | 0.154 | 0.126 | 0.887 |
| LightGBM | Light Gradient Boosting Machine | 1.789 | 6.217 | 2.492 | 0.782 | 0.154 | 0.126 | 0.885 |
| ETR | Extra Trees Regressor | 1.766 | 6.338 | 2.516 | 0.778 | 0.156 | 0.125 | 0.883 |
| RFR | Random Forest Regressor | 1.833 | 6.672 | 2.582 | 0.766 | 0.160 | 0.130 | 0.876 |
| XGBoost | Extreme Gradient Boosting | 1.856 | 6.849 | 2.616 | 0.760 | 0.162 | 0.130 | 0.873 |
| GBR | Gradient Boosting Regressor | 2.013 | 7.434 | 2.725 | 0.740 | 0.171 | 0.144 | 0.862 |
| ANN | Artificial Neural Networks | 2.022 | 7.914 | 2.812 | 0.723 | 0.181 | 0.140 | 0.860 |
| OMP | Orthogonal Matching Pursuit | 2.164 | 8.098 | 2.844 | 0.717 | 0.183 | 0.155 | 0.847 |
| AdaBoost | AdaBoost Regressor | 2.694 | 11.439 | 3.382 | 0.600 | 0.233 | 0.217 | 0.793 |
| PAR | Passive Aggressive Regressor | 2.700 | 12.231 | 3.496 | 0.571 | 0.247 | 0.194 | 0.790 |
| DTR | Decision Tree Regressor | 2.615 | 14.213 | 3.768 | 0.501 | 0.231 | 0.181 | 0.754 |
| KNN | K Neighbors Regressor | 2.708 | 15.482 | 3.932 | 0.457 | 0.241 | 0.209 | 0.758 |
| **SdWgt:** hundred-seed weight | |  |  |  |  |  |  |  |
| **MAE**: Mean Absolute Error | |  |  |  |  |  |  |  |
| **MSE**: Mean Squared Error | |  |  |  |  |  |  |  |
| **RMSE**: Root Mean Squared Error | |  |  |  |  |  |  |  |
| **R²**: R-squared (Coefficient of Determination) | |  |  |  |  |  |  |  |
| **RMSLE**: Root Mean Squared Logarithmic Error | | |  |  |  |  |  |  |
| **MAPE**: Mean Absolute Percentage Error | |  |  |  |  |  |  |  |
| **PCC**: Pearson Correlation Coefficient | |  |  |  |  |  |  |  |

| **Table S3 (R8)** The comparison of prediction performance among CatBoost, ETR, LightGBM, RFR, BR, XGBoost, HR, GBR, KNN, ANN, OMP, SVR, AdaBoost, DTR, and PAR for seven quantitative traits in soybean was conducted. The prediction performance of each method was evaluated using the MAE, MSE, RMSE, R2, RMSLE, MAPE, and PCC metrics. All models were trained using 10-fold cross-validation. | | | | | | | | |
| --- | --- | --- | --- | --- | --- | --- | --- | --- |
| **Model** | **Model description** | **MAE** | **MSE** | **RMSE** | **R2** | **RMSLE** | **MAPE** | **PCC** |
| CatBoost | CatBoost Regressor | 28.566 | 1502.820 | 38.713 | 0.585 | 0.042 | 0.030 | 0.765 |
| LightGBM | Light Gradient Boosting Machine | 29.145 | 1536.605 | 39.143 | 0.575 | 0.042 | 0.031 | 0.759 |
| ETR | Extra Trees Regressor | 27.990 | 1558.021 | 39.418 | 0.570 | 0.043 | 0.030 | 0.755 |
| RFR | Random Forest Regressor | 29.521 | 1614.816 | 40.139 | 0.554 | 0.043 | 0.031 | 0.745 |
| BR | Bayesian Ridge | 31.514 | 1654.252 | 40.618 | 0.543 | 0.044 | 0.033 | 0.737 |
| XGBoost | Extreme Gradient Boosting | 29.618 | 1689.209 | 41.037 | 0.533 | 0.044 | 0.031 | 0.734 |
| HR | Huber Regressor | 30.324 | 1712.108 | 41.331 | 0.527 | 0.045 | 0.032 | 0.730 |
| KNN | K Neighbors Regressor | 29.924 | 1768.044 | 41.976 | 0.512 | 0.045 | 0.032 | 0.718 |
| GBR | Gradient Boosting Regressor | 32.885 | 1768.278 | 42.002 | 0.511 | 0.045 | 0.035 | 0.720 |
| OMP | Orthogonal Matching Pursuit | 35.481 | 1960.829 | 44.235 | 0.458 | 0.048 | 0.037 | 0.677 |
| AdaBoost | AdaBoost Regressor | 37.260 | 2293.904 | 47.853 | 0.365 | 0.051 | 0.039 | 0.620 |
| DTR | Decision Tree Regressor | 34.790 | 3142.714 | 56.025 | 0.129 | 0.061 | 0.037 | 0.570 |
| PAR | Passive Aggressive Regressor | 44.334 | 3284.199 | 57.269 | 0.090 | 0.061 | 0.047 | 0.554 |
| SVR | Support Vector Regression | 44.989 | 3758.464 | 61.254 | -0.040 | 0.064 | 0.046 | 0.615 |
| ANN | Artificial Neural Networks | 57.174 | 6048.344 | 77.736 | -0.678 | 0.086 | 0.060 | 0.473 |
| **R8:** maturity date | |  |  |  |  |  |  |  |
| **MAE**: Mean Absolute Error | |  |  |  |  |  |  |  |
| **MSE**: Mean Squared Error | |  |  |  |  |  |  |  |
| **RMSE**: Root Mean Squared Error | |  |  |  |  |  |  |  |
| **R²**: R-squared (Coefficient of Determination) | |  |  |  |  |  |  |  |
| **RMSLE**: Root Mean Squared Logarithmic Error | | |  |  |  |  |  |  |
| **MAPE**: Mean Absolute Percentage Error | |  |  |  |  |  |  |  |
| **PCC**: Pearson Correlation Coefficient | |  |  |  |  |  |  |  |

| **Table S3 (R1)** The comparison of prediction performance among CatBoost, ETR, LightGBM, RFR, BR, XGBoost, HR, GBR, KNN, ANN, OMP, SVR, AdaBoost, DTR, and PAR for seven quantitative traits in soybean was conducted. The prediction performance of each method was evaluated using the MAE, MSE, RMSE, R2, RMSLE, MAPE, and PCC metrics. All models were trained using 10-fold cross-validation. | | | | | | | | |
| --- | --- | --- | --- | --- | --- | --- | --- | --- |
| **Model** | **Model description** | **MAE** | **MSE** | **RMSE** | **R2** | **RMSLE** | **MAPE** | **PCC** |
| CatBoost | CatBoost Regressor | 31.396 | 1969.768 | 44.360 | 0.415 | 0.064 | 0.045 | 0.645 |
| LightGBM | Light Gradient Boosting Machine | 31.967 | 2000.756 | 44.704 | 0.406 | 0.064 | 0.046 | 0.637 |
| RFR | Random Forest Regressor | 31.582 | 2012.964 | 44.848 | 0.402 | 0.064 | 0.045 | 0.635 |
| ETR | Extra Trees Regressor | 30.747 | 2056.462 | 45.328 | 0.389 | 0.065 | 0.044 | 0.627 |
| BR | Bayesian Ridge | 34.019 | 2104.462 | 45.855 | 0.375 | 0.066 | 0.048 | 0.613 |
| GBR | Gradient Boosting Regressor | 33.806 | 2117.452 | 45.996 | 0.371 | 0.066 | 0.048 | 0.614 |
| HR | Huber Regressor | 32.653 | 2146.939 | 46.320 | 0.362 | 0.066 | 0.047 | 0.605 |
| KNN | K Neighbors Regressor | 32.402 | 2186.344 | 46.737 | 0.351 | 0.067 | 0.046 | 0.602 |
| XGBoost | Extreme Gradient Boosting | 32.841 | 2186.623 | 46.736 | 0.351 | 0.067 | 0.047 | 0.607 |
| SVR | Support Vector Regression | 36.273 | 3017.631 | 54.921 | 0.103 | 0.077 | 0.051 | 0.564 |
| OMP | Orthogonal Matching Pursuit | 36.840 | 2364.951 | 48.615 | 0.297 | 0.069 | 0.052 | 0.546 |
| AdaBoost | AdaBoost Regressor | 36.067 | 2657.530 | 51.510 | 0.210 | 0.073 | 0.051 | 0.481 |
| DTR | Decision Tree Regressor | 39.851 | 3959.697 | 62.891 | -0.179 | 0.091 | 0.056 | 0.422 |
| ANN | Artificial Neural Networks | 49.123 | 4373.544 | 66.109 | -0.300 | 0.096 | 0.069 | 0.439 |
| PAR | Passive Aggressive Regressor | 51.428 | 4389.325 | 66.220 | -0.305 | 0.094 | 0.073 | 0.382 |
| **R1:** flowering date | |  |  |  |  |  |  |  |
| **MAE**: Mean Absolute Error | |  |  |  |  |  |  |  |
| **MSE**: Mean Squared Error | |  |  |  |  |  |  |  |
| **RMSE**: Root Mean Squared Error | |  |  |  |  |  |  |  |
| **R²**: R-squared (Coefficient of Determination) | |  |  |  |  |  |  |  |
| **RMSLE**: Root Mean Squared Logarithmic Error | | |  |  |  |  |  |  |
| **MAPE**: Mean Absolute Percentage Error | |  |  |  |  |  |  |  |
| **PCC**: Pearson Correlation Coefficient | |  |  |  |  |  |  |  |

| **Table S3 (protein)** The comparison of prediction performance among CatBoost, ETR, LightGBM, RFR, BR, XGBoost, HR, GBR, KNN, ANN, OMP, SVR, AdaBoost, DTR, and PAR for seven quantitative traits in soybean was conducted. The prediction performance of each method was evaluated using the MAE, MSE, RMSE, R2, RMSLE, MAPE, and PCC metrics. All models were trained using 10-fold cross-validation. | | | | | | | | |
| --- | --- | --- | --- | --- | --- | --- | --- | --- |
| **Model** | **Model description** | **MAE** | **MSE** | **RMSE** | **R2** | **RMSLE** | **MAPE** | **PCC** |
| CatBoost | CatBoost Regressor | 1.510 | 3.903 | 1.975 | 0.458 | 0.044 | 0.034 | 0.677 |
| SVR | Support Vector Regression | 1.521 | 4.008 | 2.001 | 0.444 | 0.044 | 0.034 | 0.676 |
| LightGBM | Light Gradient Boosting Machine | 1.536 | 4.012 | 2.002 | 0.443 | 0.044 | 0.035 | 0.666 |
| RFR | Random Forest Regressor | 1.532 | 4.022 | 2.005 | 0.441 | 0.044 | 0.035 | 0.665 |
| ETR | Extra Trees Regressor | 1.529 | 4.060 | 2.014 | 0.436 | 0.044 | 0.035 | 0.662 |
| BR | Bayesian Ridge | 1.600 | 4.283 | 2.069 | 0.406 | 0.046 | 0.036 | 0.638 |
| HR | Huber Regressor | 1.599 | 4.315 | 2.076 | 0.401 | 0.046 | 0.036 | 0.635 |
| GBR | Gradient Boosting Regressor | 1.610 | 4.339 | 2.082 | 0.398 | 0.046 | 0.036 | 0.635 |
| XGBoost | Extreme Gradient Boosting | 1.601 | 4.407 | 2.098 | 0.388 | 0.046 | 0.036 | 0.634 |
| KNN | K Neighbors Regressor | 1.651 | 4.691 | 2.165 | 0.349 | 0.048 | 0.037 | 0.611 |
| OMP | Orthogonal Matching Pursuit | 1.683 | 4.718 | 2.171 | 0.345 | 0.048 | 0.038 | 0.588 |
| AdaBoost | AdaBoost Regressor | 1.759 | 5.029 | 2.242 | 0.302 | 0.049 | 0.040 | 0.551 |
| DTR | Decision Tree Regressor | 2.142 | 8.058 | 2.837 | -0.120 | 0.063 | 0.048 | 0.454 |
| PAR | Passive Aggressive Regressor | 2.316 | 8.729 | 2.953 | -0.211 | 0.065 | 0.052 | 0.418 |
| ANN | Artificial Neural Networks | 2.697 | 13.294 | 3.645 | -0.850 | 0.082 | 0.061 | 0.384 |
| **protein:** protein content | |  |  |  |  |  |  |  |
| **MAE**: Mean Absolute Error | |  |  |  |  |  |  |  |
| **MSE**: Mean Squared Error | |  |  |  |  |  |  |  |
| **RMSE**: Root Mean Squared Error | |  |  |  |  |  |  |  |
| **R²**: R-squared (Coefficient of Determination) | |  |  |  |  |  |  |  |
| **RMSLE**: Root Mean Squared Logarithmic Error | | |  |  |  |  |  |  |
| **MAPE**: Mean Absolute Percentage Error | |  |  |  |  |  |  |  |
| **PCC**: Pearson Correlation Coefficient | |  |  |  |  |  |  |  |

| **Table S3 (oil)** The comparison of prediction performance among CatBoost, ETR, LightGBM, RFR, BR, XGBoost, HR, GBR, KNN, ANN, OMP, SVR, AdaBoost, DTR, and PAR for seven quantitative traits in soybean was conducted. The prediction performance of each method was evaluated using the MAE, MSE, RMSE, R2, RMSLE, MAPE, and PCC metrics. All models were trained using 10-fold cross-validation. | | | | | | | | |
| --- | --- | --- | --- | --- | --- | --- | --- | --- |
| **Model** | **Model description** | **MAE** | **MSE** | **RMSE** | **R2** | **RMSLE** | **MAPE** | **PCC** |
| SVR | Support Vector Regression | 0.937 | 1.588 | 1.259 | 0.592 | 0.070 | 0.054 | 0.774 |
| CatBoost | CatBoost Regressor | 0.943 | 1.570 | 1.252 | 0.597 | 0.069 | 0.055 | 0.773 |
| LightGBM | Light Gradient Boosting Machine | 0.955 | 1.598 | 1.263 | 0.589 | 0.070 | 0.055 | 0.768 |
| RFR | Random Forest Regressor | 0.962 | 1.639 | 1.280 | 0.579 | 0.071 | 0.056 | 0.761 |
| ETR | Extra Trees Regressor | 0.956 | 1.643 | 1.281 | 0.578 | 0.071 | 0.055 | 0.761 |
| BR | Bayesian Ridge | 0.991 | 1.691 | 1.300 | 0.565 | 0.072 | 0.057 | 0.752 |
| HR | Huber Regressor | 0.988 | 1.698 | 1.302 | 0.563 | 0.072 | 0.057 | 0.752 |
| GBR | Gradient Boosting Regressor | 1.011 | 1.761 | 1.326 | 0.548 | 0.074 | 0.059 | 0.742 |
| XGBoost | Extreme Gradient Boosting | 1.002 | 1.783 | 1.335 | 0.542 | 0.074 | 0.058 | 0.741 |
| OMP | Orthogonal Matching Pursuit | 1.046 | 1.859 | 1.363 | 0.522 | 0.076 | 0.061 | 0.723 |
| KNN | K Neighbors Regressor | 1.059 | 2.009 | 1.417 | 0.483 | 0.078 | 0.062 | 0.705 |
| AdaBoost | AdaBoost Regressor | 1.140 | 2.123 | 1.457 | 0.454 | 0.081 | 0.066 | 0.678 |
| DTR | Decision Tree Regressor | 1.335 | 3.252 | 1.803 | 0.164 | 0.100 | 0.077 | 0.590 |
| PAR | Passive Aggressive Regressor | 1.402 | 3.257 | 1.804 | 0.162 | 0.099 | 0.081 | 0.582 |
| ANN | Artificial Neural Networks | 1.392 | 3.440 | 1.854 | 0.114 | 0.102 | 0.079 | 0.605 |
| **oil:** oil content | |  |  |  |  |  |  |  |
| **MAE**: Mean Absolute Error | |  |  |  |  |  |  |  |
| **MSE**: Mean Squared Error | |  |  |  |  |  |  |  |
| **RMSE**: Root Mean Squared Error | |  |  |  |  |  |  |  |
| **R²**: R-squared (Coefficient of Determination) | |  |  |  |  |  |  |  |
| **RMSLE**: Root Mean Squared Logarithmic Error | | |  |  |  |  |  |  |
| **MAPE**: Mean Absolute Percentage Error | |  |  |  |  |  |  |  |
| **PCC**: Pearson Correlation Coefficient | |  |  |  |  |  |  |  |

| **Table S3 (Hgt)** The comparison of prediction performance among CatBoost, ETR, LightGBM, RFR, BR, XGBoost, HR, GBR, KNN, ANN, OMP, SVR, AdaBoost, DTR, and PAR for seven quantitative traits in soybean was conducted. The prediction performance of each method was evaluated using the MAE, MSE, RMSE, R2, RMSLE, MAPE, and PCC metrics. All models were trained using 10-fold cross-validation. | | | | | | | | |
| --- | --- | --- | --- | --- | --- | --- | --- | --- |
| **Model** | **Model description** | **MAE** | **MSE** | **RMSE** | **R2** | **RMSLE** | **MAPE** | **PCC** |
| CatBoost | CatBoost Regressor | 12.692 | 326.373 | 18.039 | 0.622 | 0.202 | 0.160 | 0.790 |
| ETR | Extra Trees Regressor | 12.745 | 337.173 | 18.324 | 0.610 | 0.205 | 0.160 | 0.782 |
| LightGBM | Light Gradient Boosting Machine | 13.029 | 339.126 | 18.388 | 0.607 | 0.207 | 0.165 | 0.781 |
| RFR | Random Forest Regressor | 13.127 | 353.613 | 18.773 | 0.591 | 0.210 | 0.166 | 0.770 |
| BR | Bayesian Ridge | 13.933 | 369.546 | 19.200 | 0.572 | 0.217 | 0.176 | 0.757 |
| XGBoost | Extreme Gradient Boosting | 13.519 | 370.149 | 19.211 | 0.571 | 0.215 | 0.169 | 0.760 |
| HR | Huber Regressor | 13.807 | 372.549 | 19.276 | 0.569 | 0.216 | 0.172 | 0.756 |
| GBR | Gradient Boosting Regressor | 14.300 | 392.681 | 19.788 | 0.546 | 0.223 | 0.182 | 0.742 |
| KNN | K Neighbors Regressor | 14.168 | 428.344 | 20.662 | 0.504 | 0.224 | 0.166 | 0.727 |
| ANN | Artificial Neural Networks | 14.441 | 416.441 | 20.377 | 0.518 | 0.234 | 0.178 | 0.741 |
| OMP | Orthogonal Matching Pursuit | 15.235 | 436.650 | 20.872 | 0.494 | 0.237 | 0.195 | 0.704 |
| SVR | Support Vector Regression | 17.392 | 625.645 | 24.983 | 0.277 | 0.267 | 0.211 | 0.658 |
| AdaBoost | AdaBoost Regressor | 18.840 | 579.582 | 24.060 | 0.328 | 0.291 | 0.267 | 0.654 |
| DTR | Decision Tree Regressor | 18.458 | 722.325 | 26.854 | 0.162 | 0.295 | 0.225 | 0.588 |
| PAR | Passive Aggressive Regressor | 20.757 | 750.223 | 27.369 | 0.131 | 0.336 | 0.262 | 0.582 |
| **Hgt:** plant height | |  |  |  |  |  |  |  |
| **MAE**: Mean Absolute Error | |  |  |  |  |  |  |  |
| **MSE**: Mean Squared Error | |  |  |  |  |  |  |  |
| **RMSE**: Root Mean Squared Error | |  |  |  |  |  |  |  |
| **R²**: R-squared (Coefficient of Determination) | |  |  |  |  |  |  |  |
| **RMSLE**: Root Mean Squared Logarithmic Error | | |  |  |  |  |  |  |
| **MAPE**: Mean Absolute Percentage Error | |  |  |  |  |  |  |  |
| **PCC**: Pearson Correlation Coefficient | |  |  |  |  |  |  |  |
